# Supplementary material for: A multi-level multi-product supply chain network design of vegetables products considering costs of quality: A case study
Source: PLoS One. 2024 Sep 3;19(9):e0303054. doi: 10.1371/journal.pone.0303054 (PMC11371210; doi:10.1371/journal.pone.0303054)
Supplement: S1 Table — (DOCX) [file pone.0303054.s001.docx]

Supporting Information

**Table S1: Parameters of case study network design**

| $\boldsymbol{pd}_{\boldsymbol{i}}^{\boldsymbol{n}}$**(ton)** | | | | | | **Reaction rate (%)** | | | $\boldsymbol{V}_{\boldsymbol{m}}$  **(km/ hour)** | | $\boldsymbol{cap}_{\boldsymbol{j}^{\boldsymbol{p}}}$**(ton)**  **&**  $\boldsymbol{cap}_{\boldsymbol{k}^{\boldsymbol{p}}}$ **(ton)** | | | $\boldsymbol{cap}_{\boldsymbol{m}^{\boldsymbol{p}}}$ **(ton)** | | | | | |
| --- | --- | --- | --- | --- | --- | --- | --- | --- | --- | --- | --- | --- | --- | --- | --- | --- | --- | --- | --- |
| i=1 | | i=2 | i=3 | i=4 | |  |  |  |  |  |  |  |  | m=1 | | | m=2 | | |
| n=1 | n=2 | n=1 | n=3 | n=2 | n=3 | p=1 | p=2 | p=3 | m=1 | m=2 | p=1 | p=2 | p=3 | p=1 | P=2 | P=3 | P=1 | P=2 | P=3 |
| 5000 | 1000 | 20000 | 2800 | 120 | 80 | 0.0104 | 0.00003 | 0.00006 | 80 | 100 | 3000 | 1500 | 150 | 20 | 10 | 1 | 2 | 1 | 0.1 |

**Table S1 in continue**

| $\boldsymbol{pri}_{\boldsymbol{n}}$**(US$/ ton)** | | | $\mathrm{Cpro}_{n}^{p}$***(US$/ ton)*** | | | | | | $\mathbf{Sa}_{\boldsymbol{n}^{\boldsymbol{p}}}$***(US$/ ton)*** | | | | | | | | | $\boldsymbol{Csto}$  ***(US$/ ton)*** | $\boldsymbol{Cf}_{\boldsymbol{j}}$  **&** $\mathbf{Cf}_{\boldsymbol{k}}$  **($)** |
| --- | --- | --- | --- | --- | --- | --- | --- | --- | --- | --- | --- | --- | --- | --- | --- | --- | --- | --- | --- |
|  |  |  | n=1, n=2 | | | n=3 | | | n=1 | | | n=2 | | | n=3 | | |  |  |
| n=1 | n=2 | n=3 | p=1 | p=2 | p=3 | p=1 | p=2 | p=3 | p=1 | p=2 | p=3 | p=1 | p=2 | p=3 | p=1 | p=2 | p=3 |  |  |
| 600 | 600 | 300 | 2 | 2 | 120 | 2 | 4 | 120 | 840 | 1520 | 7600 | 840 | 1520 | 7600 | 600 | 760 | 3800 | 2 | 400 |

**Source(s)**: Authors’ work
