# Supplementary material for: A multi-level multi-product supply chain network design of vegetables products considering costs of quality: A case study
Source: PLoS One. 2024 Sep 3;19(9):e0303054. doi: 10.1371/journal.pone.0303054 (PMC11371210; doi:10.1371/journal.pone.0303054)
Supplement: S2 Table — (DOCX) [file pone.0303054.s002.docx]

|  | **Table S2: Quantity (tons) of each product delivered to customers and number of vehicles in logistics of designed SC** | | | | | | | | | | | | | | | | | | | | | | | | | | | | | | | | | | | | | | | | | | | |
| --- | --- | --- | --- | --- | --- | --- | --- | --- | --- | --- | --- | --- | --- | --- | --- | --- | --- | --- | --- | --- | --- | --- | --- | --- | --- | --- | --- | --- | --- | --- | --- | --- | --- | --- | --- | --- | --- | --- | --- | --- | --- | --- | --- | --- |
| N0. Of vehicles | **Wholesalers and demand of customers (tons)-**  **(Y-R) as “Yarrow-Raw type”, (Y-D) as “Yarrow-Dried type“- (Y-E) as “Yarrow-Essence type”.**  **(B-R) as “Borage flower-Raw type”, (B-D) as “Borage flower-Dried type“- (B-E) as “Borage flower-Essence type”.**  **(M-R) as “Melisa-Raw type”, (M-D) as “Melisa-Dried type“- (M-E) as “Melisa-Essence type”.** | | | | | | | | | | | | | | | | | | | | | | | | | | | | | | **Storage center** | | | | | **Process center** | | | | | **Destination**  **Source** | | | |
|  | 80(Y-R)*- 40(Y-D)- 4(Y-E)/ 240(B-R)*- 120(B-D)- 12(B-E)/ 2(M-R)*- 1(M-D)- 0.1(M-E) | 80(Y-R)*- 40(Y-D)- 4(Y-E)/ 240(B-R) - 120(B-D)- 12(B-E)/ 2(M-R)*- 1(M-D)- 0.1(M-E) | 80(Y-R) - 40(Y-D)- 4(Y-E)/ 240(B-R) - 120(B-D)- 12(B-E)/ 2(M-R)*- 1(M-D)- 0.1(M-E) | 80(Y-R)- 40(Y-D)- 4(Y-E)/ 240(B-R)- 120(B-D)- 12(B-E)/ 2(M-R)- 1(M-D)- 0.1(M-E) | 160(Y-R)- 80(Y-D)- 8(Y-E)/ 480(B-R)- 240(B-D)- 24(B-E)/ 4(M-R)- 2(M-D)- 0.2(M-E) | 80(Y-R)- 40(Y-D)- 4(Y-E)/ 240(B-R)- 120(B-D)- 12(B-E)/ 2(M-R)- 1(M-D)- 0.1(M-E) | 80(Y-R)- 40(Y-D)- 4(Y-E)/ 240(B-R)- 120(B-D)- 12(B-E)/ 2(M-R)- 1(M-D)- 0.1(M-E) | 80(Y-R)- 40(Y-D)- 4(Y-E)/ 240(B-R)- 120(B-D)- 12(B-E)/ 2(M-R)- 1(M-D)- 0.1(M-E) | 80(Y-R)- 40(Y-D)- 4(Y-E)/ 240(B-R)- 120(B-D)- 12(B-E)/ 2(M-R)- 1(M-D)- 0.1(M-E) | 80(Y-R)- 40(Y-D)- 4(Y-E)/ 240(B-R)- 120(B-D)- 12(B-E)/ 2(M-R)- 1(M-D)- 0.1(M-E) | 80(Y-R)- 40(Y-D)- 4(Y-E)/ 240(B-R)- 120(B-D)- 12(B-E)/ 2(M-R)- 1(M-D)- 0.1(M-E) | 80(Y-R)- 40(Y-D)- 4(Y-E)/ 240(B-R)- 120(B-D)- 12(B-E)/ 2(M-R)- 1(M-D)- 0.1(M-E) | 80(Y-R)- 40(Y-D)- 4(Y-E)/ 240(B-R)- 120(B-D)- 12(B-E)/ 2(M-R)- 1(M-D)- 0.1(M-E) | 80(Y-R)- 40(Y-D)- 4(Y-E)/ 240(B-R)- 120(B-D)- 12(B-E)/ 2(M-R)- 1(M-D)- 0.1(M-E) | 80(Y-R)- 40(Y-D)- 4(Y-E)/ 240(B-R)- 120(B-D)- 12(B-E)/ 2(M-R)- 1(M-D)- 0.1(M-E) | 80(Y-R)- 40(Y-D)- 4(Y-E)/ 240(B-R)- 120(B-D)- 12(B-E)/ 2(M-R)- 1(M-D)- 0.1(M-E) | 80(Y-R)- 40(Y-D)- 4(Y-E)/ 240(B-R)- 120(B-D)- 12(B-E)/ 2(M-R)- 1(M-D)- 0.1(M-E) | 160(Y-R)- 80(Y-D)- 8(Y-E)/ 480(B-R)- 240(B-D)- 24(B-E)/ 4(M-R)- 2(M-D)- 0.2(M-E) | 80(Y-R)- 40(Y-D)- 4(Y-E)/ 240(B-R)- 120(B-D)- 12(B-E)/ 2(M-R)- 1(M-D)- 0.1(M-E) | 80( Y -R)- 40( Y -D)- 4( Y -E)/ 240(B-R)- 120(B-D)- 12(B-E)/ 2(M-R)- 1(M-D)- 0.1(M-E) | 80( Y -R)- 40( Y -D)- 4( Y -E)/ 240(B-R)- 120(B-D)- 12(B-E)/ 2(M-R)- 1(M-D)- 0.1(M-E) | 80( Y -R)- 40( Y -D)- 4( Y -E)/ 240(B-R)- 120(B-D)- 12(B-E)/ 2(M-R)- 1(M-D)- 0.1(M-E) | 80( Y -R)- 40( Y -D)- 4( Y -E)/ 240(B-R)- 120(B-D)- 12(B-E)/ 2(M-R)- 1(M-D)- 0.1(M-E) | 80( Y -R)- 40( Y -D)- 4( Y -E)/ 240(B-R)- 120(B-D)- 12(B-E)/ 2(M-R)- 1(M-D)- 0.1(M-E) | 80( Y -R)- 40( Y -D)- 4( Y -E)/ 240(B-R)- 120(B-D)- 12(B-E)/ 2(M-R)- 1(M-D)- 0.1(M-E) | 80( Y -R)- 40( Y -D)- 4( Y -E)/ 240(B-R)- 120(B-D)- 12(B-E)/ 2(M-R)- 1(M-D)- 0.1(M-E) | 320( Y -R)- 160( Y -D)- 16( Y -E)/ 960(B-R)- 480(B-D)- 48(B-E)/ 8(M-R)- 4(M-D)- 0.4(M-E) | 80( Y -R)- 40( Y -D)- 4( Y -E)/ 240(B-R)- 120(B-D)- 12(B-E)/ 2(M-R)- 1(M-D)- 0.1(M-E) | 80(Y-R)- 40(Y-D)- 4(Y-E)/ 240(B-R)- 120(B-D)- 12(B-E)/ 2(M-R)- 1(M-D)- 0.1(M-E) | 80(Y-R)- 40(Y-D)- 4(Y-E)/ 240(B-R)- 120(B-D)- 12(B-E)/ 2(M-R)- 1(M-D)- 0.1(M-E) |  |  |  |  |  |  |  |  |  |  |  |  |  |  |
|  | **30- Zahedan** | **29- Bandarabbas** | **28- Birjand** | **27- Kerman** | **26- Shiraz** | **25- Boushehr** | **24- Yasouj** | **23- Yazd** | **22- Mashhad** | **21- Bojnourd** | **20- Gorgan** | **19- Uromia** | **18- Tabriz** | **17- Ardabil** | **16- Rasht** | **15- Sari** | **14- Semnan** | **13- Isfahan** | **12- Ahvaz** | **11- Shahrekord** | **10- Ilam** | **9- Ghom** | **8- Karaj** | **7- Arak** | **6- Khoram abad** | **5- Ghazvin** | **4- Tehran** | **3- Zanjan** | **2- Sanandaj** | **1- Kermanshah** | **Asadabad** | **Nahavand** | **Malayer** | **Kaboudrahang** | **Razan** | **Asadabad** | **Nahavand** | **Malayer** | **Kaboudrahang** | **Razan** | **Transportation modes mode** | **as Farms Farm centers** | **As Processing centers** | **As storage centers** |
| 1400 tons/ 140 trailers | 40 (Y-D) | 40 (Y-D) | 40 (Y-D) | 40 (Y-D) | 80 (Y-D) | 40 (Y-D) | 40 (Y-D) | 40 (Y-D) | 40 (Y-D) | 40 (Y-D) | 40 (Y-D) | 40 (Y-D) | 40 (Y-D) | 40 (Y-D) | 40 (Y-D) | 40 (Y-D) | 40 (Y-D) | 80 (Y-D) | 40 (Y-D) | 40 (Y-D) | 40 (Y-D) | 40 (Y-D) | 40 (Y-D) | 40 (Y-D) | 40 (Y-D) | 40 (Y-D) | 160 (Y-D) | 40 (Y-D) | 40 (Y-D) | 40 (Y-D) | - | - | - | - | - | - |  | - | - | - | Trailer | **Razan** | **Razan** | **Razan** |
| 4300 tons/ 4300 trucks | 4(Y-E) | 4(Y-E) | 80(Y-R), 4(Y-E) | 80(Y-R), 4(Y-E) | 160(Y-R), 8(Y-E) | 4(Y-E) | 80(Y-R), 4(Y-E) | 80(Y-R), 4(Y-E) | 80(Y-R), 4(Y-E) | 4(Y-E) | 80(Y-R), 4(Y-E), 240(B-R) | 4(Y-E) | 4(Y-E), 240(B-R) | 4(Y-E), 240(B-R) | 80(Y-R), 4(Y-E), 240(B-R) | 80(Y-R), 4(Y-E), 240(B-R) | 80(Y-R), 4(Y-E) | 160(Y-R), 8(Y-E) | 4(Y-E) | 4(Y-E) | 80(Y-R), 4(Y-E) | 80(Y-R), 4(Y-E) | 80(Y-R), 4(Y-E), 240(B-R) | 4(Y-E) | 4(Y-E) | 80(Y-R,- 4(Y-E), 240(B-R) | 280(Y-R)-16(Y-E)- 880(B-R) | 4 (Y-E), 240 (B-R) | 4 (Y-E) | 4 (Y-E) | - | - | - |  | 1640 (F- R), 1400 (F- D)- 140 (F- E), 2800 (B-R) | - |  | - |  | 3180 (Y), 2800 (B) | Truck |  |  |  |
| 0 trailer | - | - | - | - | - | - | - | - | - | - | - | - | - | - | - | - | - | - | - | - | - | - | - | - | - | - | - | - | - | - | - | - | - | - | - | - |  | - | - | - | trailer | **Kaboudrahang** | **Kaboudrahang** | **Kaboudrahang** |
| 2280tons/ 2580 trucks |  |  |  |  |  |  |  |  |  |  |  | 80(Y-R) | 80(Y-R) | 80(Y-R) |  |  |  |  |  | 80(Y-R) |  |  |  | 80(Y-R) |  |  | 40(Y-R) | 80(Y-R) | 80(Y-R) |  | - |  | 80 (F- R) | 600 (Y-R) | - | 160 (Y) | 160 (Y) | - | 680 (Y) |  | truck |  |  |  |
|  |  |  |  |  |  |  |  |  |  |  |  |  |  |  |  |  |  |  |  |  |  |  |  |  |  |  |  |  |  |  |  |  |  | **Table S2 in continue** | | | | | | | | | | |
| 1500 tons/ 150 trailers | 120(B-D) |  | 120(B-D) | 120(B-D) |  |  |  | 120(B-D) | 120(B-D) |  |  |  |  |  |  | 60(B-D) | 120(B-D) | 240(B-D) |  |  |  | 120(B-D) | 120(B-D) | 120(B-D) |  | 120(B-D) |  |  |  |  | - | - | - | - | - | - |  | - | - | - | trailer | **Malayer** | **Malayer** | **Malayer** |
| 5117.9tons/ 5118 trucks | 12 (B-E), 0.1 (M-E) | 2 (M-R), 0.1 (M-E) | 240 (B-R), 12 (B-E), 2 (M-R), 0.1 (M-E) | 12 (B-E), 2 (M-R),0.1 (M-E) | 4 (M-R),0.2 (M-E) | 80 (Y-R), 2(M-R),0.1(M-E) | 2 (M-R),0.1 (M-E) | 240 (B-R), 12 (B-E), 2 (M-R),0.1 (M-E) | 240 (B-R), 12 (B-E), 2 (M-R),0.1 (M-E) | 2(M-R),0.1(M-E) | 2(M-R),0.1(M-E) | 2(M-R),0.1(M-E) | 2(M-R),0.1(M-E) | 2(M-R),0.1(M-E) | 2(M-R),0.1(M-E) | 6 (B-E), 2 (M-R),0.1 (M-E) | 12 (B-E), 2 (M-R),0.1 (M-E) | 440 (B-R), 24 (B-E), 4 (M-R),0.2 (M-E) | 2(M-R),0.1(M-E) | 2(M-R),0.1(M-E) | 2(M-R),0.1(M-E) | 240(B-R), 12(B-E), 2(M-R),0.1(M-E) | 12(B-E), 2(M-R),0.1(M-E) | 240(B-R), 12(B-E), 2(M-R),0.1(M-E) | 2(M-R),0.1(M-E) | 12 (B-E), 2 (M-R),0.1 (M-E) | 8(M-R),0.4(M-E) | 2(M-R),0.1(M-E) | 2(M-R),0.1(M-E) | 2 (M-R),0.1 (M-E) |  |  | 1640 (B-R), 1500(B-D),150(B-E), 68(M-R), 3.5(M-E) | - | - | - |  | 71.5 (M) | - | - | truck |  |  |  |
| 1535 tons/ 154 trailers | 1 (M-D) | 120 (B-D), 1 (M-D) | 1 (M-D) | 1 (M-D) | 240 (B-D), 2 (M-D) | 120 (B-D), 1 (M-D) | 120 (B-D), 1 (M-D) | 1 (M-D) | 1 (M-D) | 1 (M-D) | 1 (M-D) | 1 (M-D) | 1 (M-D) | 1 (M-D) | 1 (M-D) | 1 (M-D) | 1 (M-D) | 2 (M-D) | 120 (B-D), 1(M-D) | 120 (B-D), 1(M-D) | 120 (B-D), 1(M-D) | 1 (M-D) | 1 (M-D) | 1 (M-D) | 120 (B-D), 1(M-D) | 1 (M-D) | 420 (B-D), 4(M-D) | 1 (M-D) | 1 (M-D) | 1 (M-D) |  |  |  | - | - |  |  | - | - | - | trailer | **Nahavand** | **Nahavand** | **Nahavand** |
| 18170tons/ 18170 trucks |  | 240 (B-R), 12 (B-E) |  | 240 (B-R) | 480 (B-R), 24 (B-E) | 240 (B-R), 12 (B-E) | 240 (B-R), 12 (B-E) |  |  |  |  |  |  |  |  |  | 240 (B-R) | 40 (B-R) | 80 (Y-R), 240 (B-R), 12 (B-E) | 240 (B-R), 12 (B-E) | 240 (B-R), 12 (B-E) |  |  |  | 80 (Y-R), 240 (B-R), 12 (B-E) |  | 80 (B-R), 42 (B-E) |  |  | 240 (B-R) |  | 160(Y-R), 3000(B-R), 1500(B-D), 150(B-E), 35 (M-D) |  | - | - | -2040 (B) | 4650 (C), 35 (M) | 3290 (B) | - | - | truck |  |  |  |
| 1200 tons/ 120 trailers |  |  |  |  |  |  |  |  |  | 120 (B-D) | 120 (B-D) | 120 (B-D) | 120 (B-D) | 120 (B-D) | 120 (B-D) | 60 (B-D) |  |  |  |  |  |  |  |  |  |  | 60 (B-D) | 120 (B-D) | 120 (B-D) | 120 (B-D) | - | - | - | - | - | - |  | - | - | - | trailer | **-** | **Asadabad** | **Aasadabad** |
| 3200 tons/ 3200 trucks |  |  |  |  |  |  |  |  |  | 80 (Y-R), 240 (B-R), 12 (B-E) | 12 (B-E) | 240 (C—R), 12 (B-E) | 12 (B-E) | 12 (B-E) | 12 (B-E) | 6 (B-E) |  |  |  |  |  |  |  |  |  |  | 6 (B-E) | 12 (B-E) | 240 (B-R), 12 (B-E) | 80 (Y-R), 12 B-E) | -160 (Y-R), 720(B-R)- 1200(B-D), 120(B-E) | - | - | - | - | - |  | - | - | - | truck |  |  |  |

*No. of trailers needed: 564, No. of trucks needed: 33068.

*The Table has highlighted the chain’s unmet demands.

**Source(s)**: Authors’ work
