# Supplementary material for: A multi-level multi-product supply chain network design of vegetables products considering costs of quality: A case study
Source: PLoS One. 2024 Sep 3;19(9):e0303054. doi: 10.1371/journal.pone.0303054 (PMC11371210; doi:10.1371/journal.pone.0303054)
Supplement: Attachment — Submitted filename: Response to Reviewers.docx [file pone.0303054.s003.docx]

*Editor points:*

Dear editor,

Thank you for the comments which led us to make the paper ready to be sent the journal in its standards. We have done the changes in the paper as follows.

- We add authors’ information in the manuscript.
- We do the requirements of the journal in the paper to be the paper proper for the journal.
- We add data in an appendix in a supplementary file and mentioned it in a manuscript.
- We make ready three other files as, manuscript, manuscript with track changes and response to the reviewers’ comments and attached them in the revision process.

*Point-by-Point Response Letter*

**Reviewer 1:**

Dear reviewer,

Thank you for your insightful comments and suggestions which led us to improve the paper. We have modified the manuscript thoroughly according to your valuable comments and helpful suggestions. Please find the revised version of the paper enclosed. The following are our responses to your comments. Please note that the referees’ comments are written in green and our responses in black. Also, the by-one-by response to the comments are as follows.

1. It should be recommended to add exact discussion about considering linear behavior for a perishable supply chain.

Thank you for your comment. The linear behavior for a perishable agricultural products, independent of environmental factors such as temperature variations has been re-explained in the “literature review” as follows:

In practice the decrease of a single quality attribute can be approximated by one of the four basic types of mechanism which are zero order reactions having linear kinetics, Michaelis Menten kinetics, first order reactions having exponential kinetics and autocatalytic reactions with logistic kinetics (HAYAKAWA and TIMBERS, 1977; Varoquaux and Wiley, 1994). Although linear kinetics are relatively rare, Michaelis Menten kinetics are observed more frequently. The Micaelis Menten kinetics reduces to a linear one in the initial region of decay, which is the most important in quality assessment. Therefore the variable of quality in vegetables in the initial region of decay can be considered as the function of time post-harvest linearly (Tijskens and Polderdijk, 1996). It is shown in Equation 1.

$\boldsymbol{Q}\left( \boldsymbol{t} \right)\boldsymbol{=}\boldsymbol{Q}_{\boldsymbol{0}}\boldsymbol{-k} \boldsymbol{.t}$	(1)

Where, *Q_0_* is the initial quality, *t* is time *and k* is a degradation rate. In a dynamic environment, as the quality function works by the exponential kinetics, the degradation rate of quality (*k*) depends on the type of chemical synthesis, the activation energy of material and the gas constant by Arrhenius relation (Chang, 1900).

2. Some sensitivity analysis are so trivial and should be omitted.

Thank you for your comment. Two last part of the sensitivity analysis section has been improved not to be trivial as follows:

Since the increased revenue is greater than the increased cost, increasing the speed (v) will increase the chain profit; increasing the speed (v) up to 100% will increase the profit by 2.9 million USD (increased by 100%). Increasing the speed (v) will not affect the farmers’ revenue. The results comply with findings of Patidar and Agrawal in research of traditional agricultural chains in India, in which the transportation share in SC costs reached about 92% in the distribution sector (Patidar and Agrawal, 2020). It shows the importance of transportation strategies in this sector.

Regarding demands for fresh products, as shown in Figure 2, their amounts in the two cases (with and without a secondary market) are 10868 and 10068 tons, respectively, showing an increase of about 0.08 times; this leads to a quality increase and, hence, customer satisfaction and profit increase. In both cases, demands for dry and essence products are fully satisfied. The results show that new designed supply chain is applicable in the field of perishable products supply chain. It confirms the necessity of supplying innovative products of perishable ones such as processed agricultural products to meet new customer needs in a lateral market in order to the competitiveness. It complies with the findings of Malynka and Perevozova, who proposed the lateral markets in mature and immature markets in brand creation process (Malynka and Perevozova, 2019).

3. Literature review should be checked. Some significant published work has not been considered

Thank you for your comment. The related papers have been studied, hence the “Introduction” and “literature review” has been updated by relevant papers. Also, the recent quantitative models have been studied, as all are shown orderly on pages 2-5, as follows:

- In the introduction section:

Quality of vegetables is one of important measures to its customers due to quality deterioration rate of products which relates to the health of consumers (Forman et al., 2012). Time decay and shortages are a common phenomenon in products with short life cycles, and financial volatility necessitates more accurate characterization of inventory costs based on time-adjusted value (Ali et al., 2013). Moreover the quality deterioration often happens in traditional supply chains which, for the most part, are poorly planned

Recently some strategies studied in supply chain management of perishable products due to control the perishability of products which are inventory management (Ali et al., 2021), reverse logistic management (Ali et al., 2020), pricing (Mohammadi et al., 2023) and robust optimization (Goli et al., 2023)**.**

Today, lateral marketing is the most effective way of competing on the mature/immature markets, where micro-segmentation and plenty of brands don’t leave any space for new opportunities (Malynka and Perevozova, 2019).

- In the literature review section:

The optimal operation strategy is acquired based on product quality (Guo et al., 2021).

Making decisions in an integrated way will reduce costs compared to individual decisions made at each level (Salehi et al., 2020; Song and Wu, 2022), Pasha et al. studied an integrated bi-objective quality-based production-distribution agri-food MILP SC model in which profitability is maximized by defining the quality as a function of such decisions as the location of hubs and transportation strategy throughout the SC. Moreover, in the greenery SCs, De Keizer et al. presented a model on which decisions made on the greenhouse location (strategic) based on the plant’s lifetime in that location (De Keizer et al., 2017). As changes in the temperature and enthalpy levels change the food quality (Soysal et al., 2012), Khazaeli et al. and Rong et. al determined the temperature of distribution centers and deliveries so as to meet the expectations of different customers as the operational decision making in a supply chain programming (Khazaeli et al., 2023; Rong et al., 2011).

In a dynamic environment, as the quality function works by the exponential kinetics, the degradation rate of quality (*k*) depends on the type of chemical synthesis, the activation energy of material and the gas constant by Arrhenius relation (Chang, 1981). In the most of network designs, cost and benefit and quality factors are the most important factors that are to be optimized. Mostly, agri-food should make a logical balance between two topics of price reduction and customer service improvement (Soysal, Bloemhof-Ruwaard et al. 2012). Usually, the economic criteria are considered based on resource utilization and customer responsiveness; the latter means meeting part of the customer demand in due delivery time.. De Keizer et al. and Khazaeli et al. indicated quality of agricultural products causes cost in supply chain’s network designs. They showed that there is a conflict between factors of cost and quality (De Keizer et al., 2015; Khazaeli et al., 2023).

4. Comparative analysis should be covered difference between present condition of case study and proposed approach.
   Thank you for your feedback. As the following comparison between present condition of case study and proposed approach were suggested by the reviewer to be included in the paper, we have done our best to clarify this issue by Figure 10 and its descriptions as follows:

The comparison between results of present supply chain design in the case study and the results with lateral market indicates that a lateral market in the supply chain will increase the chain profit and farmer income. However, in the optimal mode in this case study, they are increasing from 9.7 and 5.85 to 12.5 (about +50%) and 8.8 (about +20%), respectively.

The results show that new designed supply chain is applicable in the field of perishable products supply chain. It confirms the necessity of supplying innovative products of perishable ones such as processed agricultural products to meet new customer needs in a lateral market in order to the competitiveness. It complies with the findings of Malynka and Perevozova, who proposed the lateral markets in mature and immature markets in brand creation process (Malynka and Perevozova, 2019).…

**Reviewer 2:**

Dear reviewer,

Thank you for the time you put in to evaluate our manuscript. Your feedback has been invaluable to our work. Please find the revised version of the paper enclosed. The following are our responses to your comments. Please note that the referees’ comments are written in green and our responses in black. Also, the by-one-by response to the comments are as follows.

Although the problem studied in this article is ok but the exposition is incomplete and inaccurate. Most of the viewpoints are simply explained about not fully explained, and there are many problems in the model setting. Simplifying the model may get better results. Based on these concerns, I have to recommend that this paper should be considered for major revision. While the paper provides a clear outline of the paper's objectives and methodology, it lacks specific details on the theories and approaches employed. To enhance the clarity and comprehensibility, I suggest incorporating the following

We appreciate your positive feedback on the topic. We have modified the manuscript according to the reviewer’s helpful comments and suggestions. We’ve done our best to more precisely clarify the confusing and poorly written component. In our opinion, now the manuscript is more suitable to the Journal.

1. Provide a brief summary of the approach: Explain the key principles and advantages of the method included to provide readers with a better understanding of its relevance to the proposed models.

Thank you for your comment. The present study -past researches relationship is shown in the “the research gap and contributions”, as follows:

A review of quantitative SC researches on the perishability of agri-food by considering quality costs in Section 2, outlines the gaps in the literature as follows:

1. Despite the importance of cost of qualities in designing SCs due to the perishability of the products, the cost of quality concept has not been widely incorporated by researchers in the design of agricultural products’ SCs.

2. No research has paid attention to the lateral market to look at the quality problems from the side in which covering some target customers.

3. Few researchers have considered benefits of several stakeholders of the agricultural SCs simultaneously. The stakeholders in agricultural Products’ SC are consumers, farmers, environment and society.

Due to the importance and necessity of developing supply chain management (SCM) from a larger perspective to provide a win-win situation for each participant in the SC, in this paper, we aim to develop a novel mathematical model in order to design a SC network, based on quality function elements in the vegetables’ sector.

The main contributions are as follows:

- Providing a network design model for an integrated multi-level SC of perishable products wherein profit is optimized by considering quality decay aspect of the products.
- Optimizing the profit of the SC of perishable products considering different quality costs for them due to unmet demand, product waste and reduced revenue of low-quality products.
- Introducing a strategy of selling perishable products to lateral markets before letting products enter the chain to prevent the production of low-quality products along it.
- Enabling the purchase of the farmer’s total agricultural product above the contract ceiling due to unpredictable production to prevent waste production and its scattering in the environment.
- Introducing a strategy of producing semi-processed, low-quality products (from those that did not enter the chain) to meet part of the market demand for lower-quality lower-price products.

The key principles and advantages of the method included to provide readers with a better understanding of its relevance to the proposed models are mentioned as follows.

The proposed SCND is a multi-product, multi-echelon model with exact (certain) demand that makes decisions at strategic, tactical and operational levels. It has focused on “quality” by considering the quality deterioration in a Micaelis Menten kinetics in the initial region of decay reduced to a linear one, moreover, by defining costs of quality degradation in the quality-cost functions.

The developed model is a four-echelon SC of perishable agricultural products in which static decade rate of the products is considered. Moreover, a lateral market is considered in the SC that does not stand higher than the vertical marketing and complete the main market. In the end, the developed model is applied to a case study of a firm in agricultural products’ industry with four echelons of farm-processing-distribution-customer centers. The vegetables selected as candidate for the present SC network design are *Yarrow*, *Borage flower* and *Melisa*, due to their priority in agricultural studies and their application in various industries (Agriculture ministry of Iran, 2022).

2. Elaborate on the multiple-objective programming tools: Highlight the specific tools and techniques utilized and how they contribute to addressing the presence of such a huge network problem. This would help the readers comprehend the novelty and significance of the approach. Research gaps can be improved by using https://doi.org/10.3390/math9172093

Although there are some researches done in order to minimize quality losses of perishable products by multi-objective problem solving approaches (Ali et al., 2021, 2020; Goli et al., 2023; Khazaeli et al., 2023; Pasha et al., 2021), the programming in present research is a single objective problem solving by profit objective function underlying quality loss costs. Hence the proposed model is not a multi-objective, but also a single objective one.

The mentioned reference is cited as a related research in other parts of the manuscript, “the literature review”.

3. Expand on the sufficient conditions to estimate the model: Provide further insights/assumptions into the conditions established by the proposed models, explain how these conditions are derived, and clarify their role in the overall analysis for the case data. Separate section to be considered for Case considerations.

Firstly, the conditions and assumptions of the modeling are presented in first part of section 3 as follows.

The designed supply chain of the firm is shown in Figure 1.

**Figure 1. Flow diagram of the agricultural products’ SC.**

Source(s): Authors’ work

First, products through related contracts and in-excess products are bought from farmers in the study area. In the second echelon of the proposed SC, some or all of the purchased products are processed at related centers resulting in different degrees of product quality. Third, the products in former echelon are stored in cool storage centers until being distributed and fourth, they are sold to wholesalers. Another part of the purchased products are transferred to the second market as lower quality products in different industries (tea bags, spices in food, etc.). Different road modes of transport are used between different echelons of the SC.

The modeling makes decisions at different echelons of the SC. Decisions made are (1) selecting farms and the quantity of raw products to be purchased from each of them, (2) the amount of products sold to the second market, (3) the number of processing and storage facilities to be settled in the SC, (4) product flow and the vehicles to carry out the transportation between the active facilities i. e. from farms to wholesalers and (5) assignment of processing facilities to the products. They are made based on minimizing the total cost of the supply chain design considering the cost of qualities. In the following, assumptions and the modeling are described.

**3.1. Assumptions**

- The location of production centers is specified.
- Capacities of the processing centers, and also storage centers are determined.
- Customer demand for each type of processed product is pre-determined.
- Shortage to customer demand is allowed.
- The deterioration rate of perishable products is not considered dynamic with environmental factors such as temperature and activation energy and the degradation rate of “k” is specific for each type of product.
- The transportation speed of each mode is assumed uniform.
- In-excess products are sold to the second market.
- Over-time quality loss-related cost, unmet customer demand and product waste are considered as quality costs.
- The cost of the lost product quality equals the price drop in proportion to the quality drop by a factor of ten^[1]^.
- The quality cost of the customer credit for each demand equals the revenue lost due to not meeting one unit demand.
- The quality cost of the product waste equals the revenue from the product sales not realized, causing that product to enter the environment as waste.
- Products are bought from farmers: 1) at a price for first-grade products based on the amount in the contract and 2) at a price for second-grade products for those over that in the contract^[2]^.
- Products are sold to the SC customers at a price for first-grade products and those outside the SC are sold in the second market at a price for second-grade products^[3]^.

After model description a separate section is set to describe the case study as follows.

In this section, we implement the proposed model on Iranian agricultural products’ firm, as a case study. Iran country has been bestowed with a wide range of climate and physio-geographical conditions and as such is most suitable for growing various kinds of vegetables, its production of vegetables is increasing. Moreover, agricultural products are profitable fields for investment. Since Iran possesses a large variety of flora with manufacturers, in equal measure, analysis of the working of the vegetable market is critical (Iranian National Standardization Organization, 2015). There is apparent shortage of related SC in Iran country. The goal of the case study is to evaluate the efficacy of the proposed model under real-world conditions and to address the needs of the firm in question. The case study used a four-echelon SCND, and materials were supplied, processed and stored (echelons 1-3) in the firm area (origin) while the last-level centers were located all over the country; in addition, a center was established as a second market to collect the in-excess products. The mentioned lateral market imposes no costs on the SC because it is closest to farms, and customers pay the transportation costs.

Suppliers were specified and contracted in advance in fertilized source centers (i=4) of selected vegetables (n=3) as a case study. There are potential processing (j=5) and storage center (k=5) candidates in the case study. The customers were trade representatives of each province all over the country (l=30). The farm centers were, in *Kaboudrahang*, *Razan*, *Nahavand* and *Malayer*. The five potential processing and storage center candidates were in *Kaboudrahang*, *Razan*, *Nahavand*, *Malayer* and *Asadabad.* The vegetable products were *Yarrow*, *Borage flower* and *Melisa*. Data were based on the 2022 statistics of the Ministry of Agriculture (Agriculture Ministry of Iran, 2022). Due to the importance of case study data publication, details of this study area are provided in Table S1 in Appendix.

4. Enhance the explanation of the application in the food sector/ perishable products: Give specific information about how the suggested models were used in the food sector/ perishable industry (please cite https://doi.org/10.1080/00207543.2012.752587), such as the data sources, variables that were looked at, and any results or insights that were gained from the use of the current data sets.

Specialty and applicability of the proposed approach in perishable products’ supply chain management is mentioned summarily at last part of “case study description section” as follows.

Generally, in presented agricultural products’ SC, some products have high quality-loss rates as well as demands for distances far from the cultivation center. This leads to a long post-harvest time for the product to reach the customer and, hence, a high rate of quality loss and a drop in the product price. This fact make the SC decision maker set the lateral market due to not deliver those products to those customers and hence deliver them to second market. It is considered newly in the present research due to make quality loss of products in the supply chain, the less, hence the profit the more.

As the reference mentioned above is in relation with other strategies of supply chain management of time-related decay rate of perishable products, it is cited in the “introduction” section as follows.

Time decay and shortages are a common phenomenon in products with short life cycles, and financial volatility necessitates more accurate characterization of inventory costs based on time-adjusted value (Ali et al., 2013).

5. The results of the paper are simply stated without good explanations. The discussion of the obtained results must be well improved, highlighting the insights of the research findings and support from earlier literature such as <https://doi.org/10.1038/s41598-022-26449-8>.

# Thank you for your comment. The results were reviewed and more completed.

# In results section, firstly the results of the approach in the present research in relation with recent researches are indicated as:.

As shown in Fig. 2, in the optimum point of maximizing profit by considering quality costs in perishable vegetables SC in the proposed MILP model, the processing, storage and distribution centers are settled in a similar locations, spatially. It leads to set process-storage-transfer type of hub centers. This is in compliance with the SC network proposed by Khazaeli et al. (Khazaeli et al., 2023). Out of 5 potential processing and storage/transfer centers, the model found all for the SC. No. of facilities based on the center capacity and its setup costs (related parameters are listed in Appendix-Table S1). It is similar to the model proposed by De Keizer et al., in which to decrease transport time and hence decay in related SC design, the centers were decentralized, (De Keizer et al., 2017), hence more hubs were opened.

# Next, parameters to which model responses investigated in reaction, are the reaction rate of products and speed of different transportation modes as they relate to the quality loss of products and cost of SC during the time after harvest. Model responses to changes have been analyzed and explained orderly in sensitivity analysis section.

At last part of results section, the comparison between present results in proposed approach with approaches in recent related researches are claimed as follows.

The results comply with findings of Patidar and Agrawal in research of traditional agricultural chains in India, in which the transportation share in SC costs reached about 92% in the distribution sector (Patidar and Agrawal, 2020). It shows the importance of transportation strategies in this sector.

Comparison of designed SC with traditional SC in the case study is demonstrated in Figure 10.

Comparison of Designed SC with SC without Lateral Market

|  | With Second Market | Without Second Market |
| --- | --- | --- |
| Farmers Revenue | 12.5 | 9.7 |
| Supply Chain Benefit | 8.8 | 5.85 |
| Fresh Products Demand Met | 10868 | 10068 |
| Dried Products Demand met | 5635 | 5635 |
| Essence Products Demand met | 563 | 563 |

*Figure 2. Designed SC with a second market in comparison with SC without a second market.*

Source(s): Authors’ work

Regarding demands for fresh products, as shown in Figure 10, their amounts in the two cases (with and without a secondary market) are 10868 and 10068 tons, respectively, showing an increase of about 0.08 times; this leads to a quality increase and, hence, customer satisfaction and profit increase. In both cases, demands for dry and essence products are fully satisfied. The comparison between results of present supply chain design in the case study and the results with lateral market indicates that a lateral market in the supply chain will increase the chain profit and farmer income. However, in the optimal mode in this case study, they are increasing from 9.7 and 5.85 to 12.5 (about +50%) and 8.8 (about +20%), respectively.

The results show that new designed supply chain is applicable in the field of perishable products supply chain. It confirms the necessity of supplying innovative products of perishable ones such as processed agricultural products to meet new customer needs in a lateral market in order to the competitiveness. It complies with the findings of Malynka and Perevozova, who proposed the lateral markets in mature and immature markets in brand creation process (Malynka and Perevozova, 2019).

6. It would be better if providing a more complete process instead of just states how to calculate it. This submission looks like the student’s assignment and not a research paper.

Thank you for your comment. The research framework has been re-written and placed in section 3 as Problem description and formulation. Moreover, the designed supply chain of the firm is shown in Figure 1 as follows.

**Figure 3. Flow diagram of the agricultural products’ SC.**

Source(s): Authors’ work

The SC is describes as follows.

First, products through related contracts and in-excess products are bought from farmers in the study area. In the second echelon of the proposed SC, some or all of the purchased products are processed at related centers resulting in different degrees of product quality. Third, the products in former echelon are stored in cool storage centers until being distributed and fourth, they are sold to wholesalers. Another part of the purchased products are transferred to the second market as lower quality products in different industries (tea bags, spices in food, etc.). Different road modes of transport are used between different echelons of the SC.

The modeling makes decisions at different echelons of the SC. Decisions made are (1) selecting farms and the quantity of raw products to be purchased from each of them, (2) the amount of products sold to the second market, (3) the number of processing and storage facilities to be settled in the SC, (4) product flow and the vehicles to carry out the transportation between the active facilities i. e. from farms to wholesalers and (5) assignment of processing facilities to the products. They are made based on minimizing the total cost of the supply chain design considering the cost of qualities.

Next, assumptions and the modeling were described respectively in sections 3-1 and 3-2.

After applying the model in a case study to investigate the validity of the proposed model, in order to necessity of applying viewpoints of considering cost of quality in the SCND of perishable products and second market settling besides the main one in the SC design, it is declared:

In presented perishable agricultural products’ SC, some products have high quality-loss rates as well as demands for distances far from the cultivation center. This leads to a long post-harvest time for the product to reach the customer and, hence, a high rate of quality loss and a drop in the product price. This fact make the SC decision maker set the lateral market due to not deliver those products to those customers and hence deliver them to second market.

7. Limitation of the work to be included along with the future research directions..

Thank you for your comments. Three research limitations, of which the last one is about solution method, and related suggestion in future researches are presented in section 5 (Conclusions) as follows:

The limitation and delimitation of the research process are as follows:

(1) Pricing policies are not considered to more maximize the profit of stakeholders.
(2) As it is computed the optimal solution by the exact-type solving method of mathematical programming with certain parameters it does not work for the problem in uncertain conditions.
(3) Due to the size of the case study problem and related certain parameters, this solution method is not applicable for large-size problems.

Hence, suggestions for further studies on this topic are as follows:

(1) Applying pricing policies for products sold to the second market.
(2) Use mathematical models to consider uncertainties in the SC parameters.
(3) Applying meta-heuristic methods to solve medium and large-sized problems.

1. The coefficient (10) is proposed by experts based on pairwise comparisons of cost and quality criteria.
2. According to experts, the purchase-price-drop coefficient is 0.3 in the market.
3. According to experts, the sell-price-drop coefficient is 0.3 in the market.
