# Supplementary material for: A multi-level multi-product supply chain network design of vegetables products considering costs of quality: A case study
Source: PLoS One. 2024 Sep 3;19(9):e0303054. doi: 10.1371/journal.pone.0303054 (PMC11371210; doi:10.1371/journal.pone.0303054)
Supplement: Attachment — Submitted filename: Response to Reviewers2.docx [file pone.0303054.s004.docx]

*Point-by-Point Response Letter*

**Reviewer 1:**

Dear reviewer,

Thank you for your insightful comment which led us to improve the paper. We have modified the manuscript thoroughly according to your valuable comment. Please find the revised version of the paper enclosed. In newly enclosed revised version of the paper, the new changes in this second revised version of the manuscript are highlighted in yellow color. Track changes show the changes in the first revision of the manuscript.

The following is our response to your comment. Please note that the referees’ comment is written in green and our responses in black.

1. Respond to first comment "It should be recommended to add exact discussion about considering linear behavior for a perishable supply chain" is not acceptable because used references are old.

Thank you for your comment. The linear behavior for perishable agricultural products, independent of environmental factors such as temperature variations has been re-explained in the “literature review” with some new references as follows:

The quality function of perishable agricultural products can be either complex or simple (Centobelli et al., 2021). It has been shown that, the decrease of a single quality attribute of agricultural products can be approximated by one of the four basic types of mechanism which are zero-order reactions having linear kinetics, Michaelis Menten kinetics, first-order reactions having exponential kinetics, and autocatalytic reactions with logistic kinetics (HAYAKAWA and TIMBERS, 1977; Varoquaux and Wiley, 1994). For the concept of keeping quality, the actual mechanism does not play a role. For this reason, it is convenient to assume zero-order reaction kinetics (Tijskens and Polderdijk, 1996), and mostly the Michaelis Menten kinetics reduces to a linear one in the initial region of decay, which is the most important in quality assessment (Hertog et al., 2014). Therefore, the variable of quality in vegetables in the initial region of decay can be considered as the function of time post-harvest linearly, which is shown in Equation 1.

$\boldsymbol{Q}\left( \boldsymbol{t} \right)\boldsymbol{=}\boldsymbol{Q}_{\boldsymbol{0}}\boldsymbol{-k} \boldsymbol{.t}$	(1)

Where, *Q_0_* is the initial quality, *t* is time *and k* is a degradation rate. Borghi D de Freitas et al. minimized the losses of fruits and vegetables that happen during its storage in a mathematical model, based on Equation 1 (Ferreira et al., 2005).

**Reviewer 2:**

Dear reviewer,

Thank you for the time you put in to evaluate our manuscript. Your feedback has been invaluable to our work. Please find the revised version of the paper enclosed. In newly enclosed revised version of the paper, the new changes in this second revised version of the manuscript are highlighted in yellow color. Track changes show the changes in the first revision of the manuscript.

The following are our responses to your comments. Please note that the referees’ comments are written in green and our responses in black. Also, the by-one-by response to the comments are as follows.

1. Consider adding an Implications section that zeroes in on managerial and policy-maker implications, as this may enhance the practical relevance of the research.

Thank you for your comment. To enhance the practical relevance of the research, the managerial implications section is added as Section 6 to the end of the manuscript, as follows:

The proposed model is generic and can help logistic managers as a tool to assist in decision-making related to the logistics of perishable agricultural products in the supply chain. Specially, the research done can have the following applications:

- A second market besides the chain and not higher than the vertical one in supply-based products such as vegetables, may result in a considerable increase in the chain profit without changing the resources, no reduction in the farmer income for unpredictable amounts of agricultural products production, and no wasted products preventing the environment from being polluted.
- The usage of lateral marketing is relevant, as it is the most effective way of competition in mature markets.
- When chains are designed for perishable products for optimum profit, the demand for some products with high quality-loss rates is not met due too long distances from distribution centers (if it is met, high-quality costs will be imposed on the chain).
- Increased perishability rate of agricultural products reveals the effects and necessity of second markets next to the chain.
- Although, high speed shipping fleets are expensive, using them will increase the chain profit because they reduce the post-harvest travel time and, hence, reduce the quality-loss-related costs of perishable products significantly. This way, the demands of more customers are met, customer credit costs will be prevented and the supply chain management and customers will both be benefitted. By applying the proposed model in the perishable agricultural products supply chain, the products are sold in the second market to meet the lateral part of the market. As a result, different stakeholders such as farmers, customers, the environment, and the owner of the supply chain may benefit from the new supply chain network design

2. A dedicated section for Future Research and Limitations can provide readers with a clearer understanding of potential avenues for further inquiry and the boundaries of the current study.

A separate section has been dedicated to future research and limitations to provide readers with a clearer understanding of potential avenues for future research and the boundaries of the current study in Section 7 as follows:

The limitations of this study include not considering pricing policies. The proposed solution method computed the optimal solution by the exact-type solving method of mathematical programming with certain parameters and it does not work for the problem in uncertain conditions. Due to the size of the case study problem and related certain parameters, this solution method is not applicable to large-size problems. Considering the limitations of and the results obtained in this study, directions for future research include considering pricing policies for products sold to the second market to maximize the profit of the stakeholders. As it is computed the optimal solution by the exact-type solving method of mathematical programming with certain parameters, using mathematical models to consider uncertainties in the SC parameters is suggested. Moreover, applying meta-heuristic methods to solve medium and large-sized problems is suggested in future research.

3. Refraining from using bullets, especially in Future research, limitations, and uniqueness of the study can help maintain a consistent and professional format throughout the manuscript.

Thank you for your comment. We refrain from using bullets in future research and limitations.

4. Why Quality as a keyword is used in the title? Replace with some other word.

Thank you for your comment. The word “Quality” in the title is not a separate word and it is part of the concept of “Cost of quality”, which is introduced by Feigenbaum and Crozby in the Total Quality Management philosophy. “Cost of quality” concept is a technique to measure the non-conformance with quality features. To the above-mentioned concept be clearer, the title is modified as follows.

“A Multi-level Multi-Product Supply Chain Network Design of Vegetables Products Considering Costs of Quality: A Case Study”.
