# Supplementary material for: A multi-level multi-product supply chain network design of vegetables products considering costs of quality: A case study
Source: PLoS One. 2024 Sep 3;19(9):e0303054. doi: 10.1371/journal.pone.0303054 (PMC11371210; doi:10.1371/journal.pone.0303054)
Supplement: Attachment — Submitted filename: Response to Reviewers-R3.docx [file pone.0303054.s005.docx]

*Point-by-Point Response Letter*

**Academic Editor:**

Dear academic editor,

Thank you for your attention to our manuscript. As you emphasized on the second reviewer questions, the questions you mentioned are answered in pages 3-4.

**Reviewer 1:**

Dear reviewer,

Thank you for your insightful comment which led us to improve the paper. We have modified the manuscript thoroughly according to your valuable comment. Please find the revised version of the paper enclosed. In newly enclosed revised version of the paper, the new changes in this third revised version of the manuscript are highlighted in yellow color. Track changes show the changes in the second revision of the manuscript.

The following is our response to your comment. Please note that the referees’ comment is written in green and our responses in black.

1- Respond of authors about linear behavior for a perishable supply chain is not suitable nowadays thence originality of work is weak.

Thank you for your comment. The linear behavior for perishable agricultural products is widely used in the literature in modeling of the products’ perishability. It has been re-explained in the “literature review”, precisely. Moreover, some new references, which uses this modeling approach in the quality definition of perishable agricultural products are mentioned to demonstrate that the application of the linear behavior of the perishable products’ quality in time has been recently used in different mathematical modeling of quality behavior, as follows:

The quality function of perishable agricultural products can be either complex or simple [44]. It has been shown that, the decrease of a single quality attribute of agricultural products can be approximated by one of the four basic types of mechanism which are zero-order reactions having linear kinetics, Michaelis Menten kinetics, first-order reactions having exponential kinetics, and autocatalytic reactions with logistic kinetics [45], [46]. For the concept of keeping quality, it is convenient to assume zero-order reaction kinetics [47], and mostly the Michaelis Menten kinetics reduces to a linear one in the initial region of decay, which is the most important in quality assessment [48]. Therefore, the quality variable of vegetables in the initial region of decay can be considered in a widely used equation, in which the quality function changes by the time linearly. It is shown in Equation 1.

$\frac{\boldsymbol{dQ}}{\boldsymbol{dt}}=\boldsymbol{k}$	$\boldsymbol{Q}\left( \boldsymbol{t} \right)=\boldsymbol{Q}_{\mathbf{0}}-\boldsymbol{k} .\boldsymbol{t}$	(1)

Where, Q0 is the initial quality, t is time and k is a degradation rate. In a dynamic environment, the well-known Arrhenius equation shows that the degradation rate (k) depends on the activation energy of the material, and the environmental factors [47], [49], [50].

The perishable products’ quality model shown in Equation 1 has been frequently used to capture the degradation of food products over time. For example, in the grocery retail chain, Wang and Li presented a pricing model to maximize food retailer’s profit in a dynamically identified food shelf life by using Equation 1 [51]. Chen and Chen proposed an on-site direct-sale dynamic supply chain inventory model, considering time-dependent quality losses for perishable foods [22]. Lejarza and Baldea presented a closed-loop, feedback-based control framework, that employs real-time product quality measurements for optimal supply chain management [52]. Moreover, Xu et al. presented a real time decision support framework to mitigate the quality degradation in the journey of agricultural perishable products from farm to the retailer in the supply chain based on the Equation 1 [53].

**Reviewer 2:**

Dear reviewer,

Thank you for the time you put in to evaluate our manuscript. Your feedback has been invaluable to our work. Please find the revised version of the paper enclosed. In newly enclosed revised version of the paper, the new changes in this second revised version of the manuscript are highlighted in yellow color. Track changes show the changes in the second revision of the manuscript.

The following are our responses to your comments. Please note that the referees’ comments are written in green and our responses in black. Also, the by-one-by response to the comments are as follows.

1. The abstract needs to have a smooth flow in terms of the discussions. Don't include pointwise discussions.

Thank you for your comment. To make the abstract have a smooth flow, especially in terms of the discussion, we re-write the abstract, as follows:

Effective logistics management is crucial for the distribution of perishable agricultural products to ensure they reach customers in high-quality condition. This research examines an integrated, multi-echelon supply chain for perishable agricultural goods. The supply chain consists of four stages: supply, processing, storage, and customers. This study investigates the quality-related costs associated with product perishability to maximize supply chain profitability. Key factors considered include the network design, location of processing and distribution centers, the ability to process raw products to minimize post-harvest quality degradation, the option to sell the excess produce to a secondary market due to unpredictable yields, and the decision not to fulfill demand from distant customers where significant quality loss and price drops would be involved, instead diverting those products to the aforementioned secondary market. Quantitative methods and linear mathematical programming are employed to model and validate the proposed supply chain using actual data from a real-world case study on vegetable supply chains. The main contribution of this research is the incorporation of quality costs into the objective function, which allows the supply chain to prioritize meeting nearby customers' demands with minimal quality loss over serving distant customers where high quality loss is unavoidable. Additionally, deploying a faster transportation fleet can significantly improve the overall profitability of the perishable product supply chain

2. Many subsections were included, which prevented the readers from having a smooth reading. Kindly adjust and remove the subsection numbers.

Thank you for your comment. The subsection numbers are made to only one and second level. The third level of the subsection numbers is removed, as it is apparent in the manuscript. Please check it in the new one.

3. Data availability references and tables/data-based in-depth information are not given.

Thank you for your comment. The more precise data availability references and data-based in-depth information are stated in the case study section, as follows:

In this section, we implement the proposed model in an Iranian raw and processed vegetable products’ company, the Razian Company, as a case study.

The case study used a four-echelon SCND, and materials were supplied, processed, and stored (echelons 1-3) in the firm area (origin) while the last-level centers were located all over the country; in addition, a center was established as a second market to collect the in-excess products, as shown in Fig. 1. The mentioned lateral market imposes no costs on the supply chain because it is closest to farms, and customers pay the transportation costs.

At first, the firm seasonally provided the vegetables from the suppliers. Suppliers were specified and contracted in advance in fertilized source centers (i=4) of selected vegetables (n=3). The farm centers were, in Kaboudrahang, Razan, Nahavand, and Malayer, and the vegetable products were Yarrow, Borage flower, and Melisa. Secondly, the firm used the related processing on vegetables, or the products remained raw. There are potential processing center (j=5) candidates in the case study. Thirdly, the firm stored the products in the storage centers for packaging. There are potential storage center (k=5) candidates in the case study. The five potential processing and storage center candidates were *Kaboudrahang*, *Razan*, *Nahavand*, *Malayer*, and *Asadabad.* Finally, the firm delivered the demanded products to the customer centers. The customers were trade representatives of each province all over the country (l=30). Due to the importance of the case study data for the application of the presented model, some were obtained from the enterprise resource planning (ERP) of Razian company (https://razian.co/). In addition, data on fixed and variable costs of different transportation modes were obtained from the recent case study research done in Iran [39]. Data on the price of different raw and processed vegetable products were gathered from the statistics of the Ministry of Agriculture [56]. Details of the most critical data of the case study are presented in Table S1 in the Appendix.

Of course, the Appendix is a separate file attached.

4. The paper needs clearer insights into future trends and the ability of researchers to pursue them based on existing research. More detailed discussions on future research directions, trends, challenges, and opportunities are needed.

Thank you for your comment. As you mentioned in this valuable comment, we re-write the limitations and future research section, with a more detailed discussion on the future research directions and opportunity to do valuable research in the future for those whom are passionate about the field of agricultural products supply chain design, as follows:

Our framework is limited in some respects. With that said, this modeling limitations serve as a platform for extending it in future researches. One primary limitation of the presented model is that it does not consider the uncertainty in the amount of customers’ demand. Therefore, the proposed model does not work for the problem in uncertain conditions. Also, the proposed model in this research has been solved by the exact-type solving method of mathematical programming, which is proper for solving the small size of problems such as the studied case. Considering the limitations above, using mathematical models by uncertainty considerations in the supply chain parameters and applying meta-heuristic methods to solve medium and large-sized problems are suggested in the future research. From the managerial perspective, the presented research works by the assumption of that upstream suppliers, freight transportation, processing centers, and storage facilities are integrated and it needs to build alignment between their organizations to deploy the solutions proposed by the output of the proposed framework. For these efforts to be successful, for future research, it is suggested to study how to cooperate all parties involved in the supply chain, and design the coordination infrastructure in the supply chain to yield the positive effects of proposed supply chain network design, in practice.

**Reviewer 3:**

Dear reviewer,

Thank you for the time you put into evaluating our manuscript. We are grateful to you for your positive feedback to our work.
